# Mechanistic modelling of dynamic MRI data predicts that tumour heterogeneity decreases therapeutic response

**DOI:** 10.1038/sj.bjc.6605773

**Published:** 2010-07-13

**Authors:** R Venkatasubramanian, R B Arenas, M A Henson, N S Forbes

**Affiliations:** 1 Department of Chemical Engineering, University of Massachusetts, 159 Goessmann Hall, 686 North Pleasant Street, Amherst, MA 01003, USA; 2 Pioneer Valley Life Sciences Institute, Springfield, MA 01107, USA; 3 Department of Surgery, Baystate Medical Center/Tufts University School of Medicine, Springfield, MA 01199, USA

**Keywords:** dynamic contrast-enhanced magnetic resonance imaging, tumour growth model, therapeutic response, predictive multiscale model, tumour heterogeneity

## Abstract

**Background:**

Dynamic contrast-enhanced magnetic resonance imaging (DCE-MRI) contains crucial information about tumour heterogeneity and the transport limitations that reduce drug efficacy. Mathematical modelling of drug delivery and cellular responsiveness based on underutilised DCE-MRI data has the unique potential to predict therapeutic responsiveness for individual patients.

**Methods:**

To interpret DCE-MRI data, we created a modelling framework that operates over multiple time and length scales and incorporates intracellular metabolism, nutrient and drug diffusion, trans-vascular permeability, and angiogenesis. The computational methodology was used to analyse DCE-MR images collected from eight breast cancer patients at Baystate Medical Center in Springfield, MA.

**Results:**

Computer simulations showed that trans-vascular transport was correlated with tumour aggressiveness because increased vessel growth and permeability provided more nutrients for cell proliferation. Model simulations also indicate that vessel density minimally affects tissue growth and drug response, and nutrient availability promotes growth. Finally, the simulations indicate that increased transport heterogeneity is coupled with increased tumour growth and poor drug response.

**Conclusion:**

Mathematical modelling based on DCE-MRI has the potential to aid treatment decisions and improve overall cancer care. This model is the critical first step in the creation of a comprehensive and predictive computational method.

The ability to predict the response of a patient to chemotherapeutic treatment is necessary to identify correct therapeutic regiments and dosages (Lonning, 2007; Zahra *et al*, 2007). In breast cancer, for example, the effectiveness of neoadjuvant chemotherapy varies dramatically among patients (Chuthapisith *et al*, 2006). Determining which patients will respond to therapy would prevent unnecessary treatment and diminish life-threatening delays until surgery. Two primary factors that reduce the efficacy of chemotherapeutics are limited delivery and reduced cellular responsiveness, both of which are associated with tumour heterogeneity (Jain, 1998; Minchinton and Tannock, 2006). Abnormal and inadequate vasculature coupled with diffusion limitation results in reduced availability of nutrients, for example glucose and oxygen (Jain, 1988; Vaupel *et al*, 1989). Cells located away from vasculature do not traverse through the cell cycle, become quiescent, and are less susceptible to cytotoxic drugs (Gardner, 2000; Kim and Tannock, 2005). The effectiveness of chemotherapeutic drugs is further reduced by inadequate delivery and limited diffusion through different microenvironments (Jain, 1999; Tannock *et al*, 2002; Minchinton and Tannock, 2006).

Information necessary to quantify therapeutic resistance can be obtained by dynamic contrast-enhanced magnetic resonance imaging (DCE-MRI), which measures the accessibility of small molecules to different tumour regions (Tofts, 1997; Turetschek *et al*, 2004; Feng *et al*, 2008). In gadopentetic acid (Gd-DTPA)-based DCE-MRI, contrast agent is injected i.v. and signal enhancement is measured as a function of location and time. Following injection, Gd-DTPA fills the vascular space, diffuses into the surrounding tissue, and is eventually washed out. Vasculature density and permeability are calculated from local time profiles using multi-compartment models (Tofts and Kermode, 1991; Tofts, 1997; Harrer *et al*, 2004). DCE-MRI has the potential to be a powerful tool for predicting therapeutic responsiveness (Sorensen, 2006) because it is in common use and is readily available (Chen *et al*, 2006; Ah-See *et al*, 2008). In previous clinical studies DCE-MRI measurements have been shown to correlate with change in tumour volume (Martincich *et al*, 2004; Hsiang *et al*, 2007) and with responsiveness to neoadjuvant chemotherapy (Padhani *et al*, 2006; Loo *et al*, 2008). Specifically, the transvascular transport parameter (*K*^trans^) has been shown to correlate with therapeutic responsiveness (Hayes *et al*, 2002; Ah-See *et al*, 2008). In addition, previous studies have shown that DCE-MRI measurements correlate with microvascular density (Hawighorst *et al*, 1997; Su *et al*, 2003). It has also been suggested that DCE-MRI could be used to indicate the efficacy of anti-angiogenic therapy (Hylton, 2006). Changes in *K*^trans^, measured 1 day after treatment with cediranib, an anti-angiogenic therapy, have been shown to predict overall and progression-free survival (Sorensen *et al*, 2009). Recently, it has also been shown that the heterogeneity of masked wavelet decomposition coefficients in DCE-MR images is a strong predictor of responsiveness to radiation treatment (Prescott *et al*, 2010) and that MRI parameters for perfusion heterogeneity and tumour volume are better predictors of tumour recurrence and death than clinical prognostic factors (Mayr *et al*, 2010).

To obtain predictions for the therapeutic response of individual patients, one needs to interpret spatially dependant transport information from DCE-MRI. Mathematical models that include molecular transport, cell growth, and drug response have the potential to interpret DCE-MRI data and provide a basis for patient-specific predictions. Previous mathematical models have predicted tumour growth rate based on the mechanisms of nutrient diffusion and cell growth and death (Adam and Maggelakis, 1990; Ward and King, 1997; Araujo and McElwain, 2004). Tumour growth models have also been used to study the effects of drug diffusion on tumour growth (Jackson and Byrne, 2000; Bertuzzi *et al*, 2003). Other more specific mathematical models have, for example, predicted the effects of vascular normalisation by anti-angiogenic drugs (Jain *et al*, 2007) and the effects of paclitaxel transport on cancer cells (Kuh *et al*, 2000). In previous work we built upon these models to show how multiple nutrients interact to control cell growth and death (Venkatasubramanian *et al*, 2006) and how transport properties dictate the efficacy of cancer therapeutics (Venkatasubramanian *et al*, 2008).

Here we describe a mathematical framework that incorporates DCE-MRI data into a tumour growth model to predict therapeutic response. We have used this computational method to test the hypothesis that local transport properties can predict therapeutic response. Specifically, we hypothesise that (1) increased trans-vascular transport predicts improved theoretical response, and (2) increased spatial heterogeneity predicts reduced theoretical response. The drug response model is based on our previous tumour models that integrate intracellular metabolism, nutrient and drug diffusion, cell-cycle progression, cellular drug effects, and drug pharmacokinetics (Venkatasubramanian *et al*, 2006, 2008). The current model integrates information on local vascular permeability and vessel density derived from DCE-MRI. The model operates over multiple length scales, including cellular (*μ*m), vascular (100 *μ*m), voxel (mm), and whole tumour (cm); and multiple time scales, including cellular diffusion (seconds), gadolinium infusion (minutes), and tumour drug response (weeks). The model was used to analyse DCE-MR images and generate predictions for eight patients at Baystate Medical Center in Springfield, MA. Because of its mechanistic basis, we believe that this theoretical framework has potential to be a predictive therapeutic tool for individualised therapy.

## Materials and methods

Included in this study were eight female breast cancer patients who were referred to the Baystate Breast MRI Center between March 2006 and May 2007. Patients, with ages ranging from 40 to 73, were selected to include a diverse population based on tumour staging and aggressiveness. The imaging procedure consisted of (1) scanning each patient to establish an unenhanced image, (2) administering 0.1 mmol kg^−1^ Gd-DTPA, and (3) scanning an additional six times, with a spacing of approximately 2 min. Ethical approval for the study was granted by the institutional review board at Baystate Health Systems (IRB07-013). All data were acquired before the study as part of standard care procedures and identifying information was removed to protect patient confidentiality (as per 45 CFR 46.101(b)). The study followed all HIPAA guidelines.

**Figure 1:**
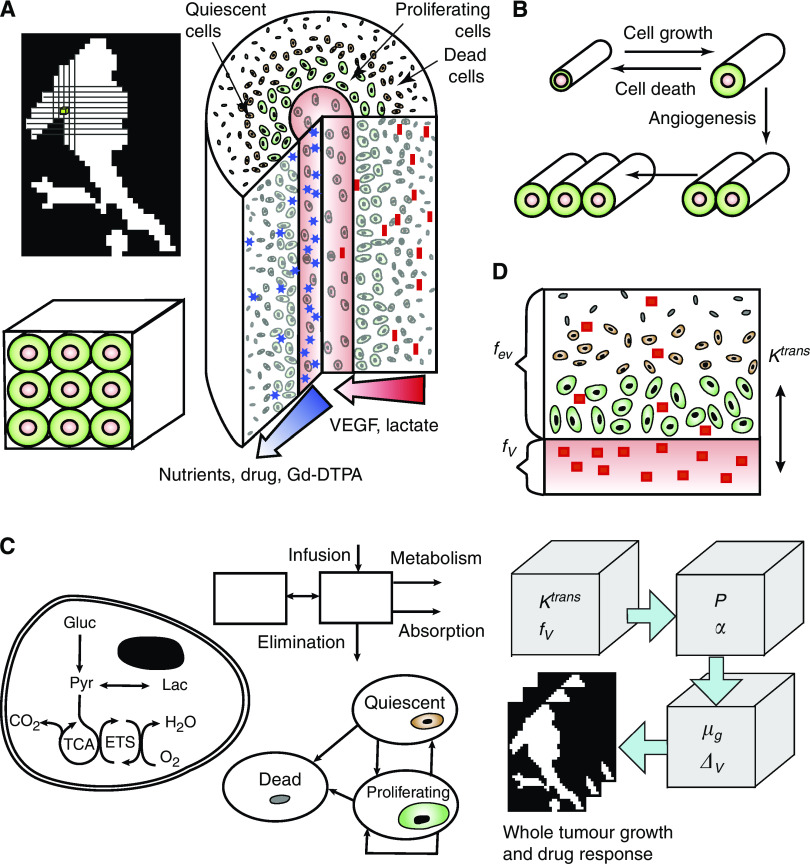
Components of tumour growth and drug response model. (**A**) The model framework incorporates four length scales: whole tumours, MRI voxels, vascular cords, and individual cells. Tumours contain a collection of cuboid voxels, whose size was fixed by spectrometer resolution. Each voxel contains a series of identical tumour cords, each consisting of tumour cells surrounding a central blood vessel. Nutrients (glucose, oxygen), anti-cancer agents, and MRI contrast agents diffuse from the vessel outwards, whereas lactate and VEGF generated in the cord exterior diffuse towards the vessel. Cell growth rate is variable with radius. Cell types typically arrange with proliferating cells close to the vessel, dead cells towards the periphery, and an intermediate region of quiescent cells. (**B**) Two processes control tumour growth: increase in cord size and increase in cord number (angiogenesis). Cord radius increases due to cell growth and decreases due to death from nutrient depletion or drug cytotoxicity. (**C**) Three additional biological components are incorporated in the model framework: intracellular metabolism, cell growth dynamics, and drug pharmacokinetics. The metabolic component integrates three nutrients: glucose, oxygen, and lactate, through glycolysis and the TCA cycle. The drug pharmacokinetic component contains two compartments and rates for infusion, metabolism, absorption, and elimination. The cell growth component contains transition rates between three cell types: proliferating, quiescent, and dead cells. Proliferating and quiescent cells die in low-nutrient or high-drug conditions. Proliferating cells either replicate or become quiescent depending on nutrient availability. (**D**) A two-compartment pharmacokinetic model with a mass transfer coefficient, *K*^trans^, describes Gd-DTPA transport between vascular (*f*_V_) and extra-vascular (*f*_ev_) spaces. A four-step process converts spatially dependant transport information from DCE-MRI into tumour predictions: (1) a *K*^trans^ and *f*_V_ parameter pair is measured for each MRI voxel; (2) each (*K*^trans^, *f*_V_) parameter pair is converted into permeability (*P*) and angiogenic sensitivity (*α*) using parameter maps based on simulation results; (3) subsequent (*P*, *α*) parameter pairs are similarly converted into local growth rate (*μ*_G_) and drug response (Δ*V*); and (4) local responses are summed to predict overall growth rate and drug responses for entire tumours.

The procedure for determining therapeutic response from DCE-MRI contained three major steps. First, a theoretical framework was developed and simulations were run to generate relations between transport parameters and local drug response (Figure 1). Second, local transport parameters were determined for each stack of MR images. Finally, local responses were calculated and summed to determine the overall response of each tumour.

### Model framework

To mechanistically interpret MRI data, the model integrated four length scales: whole tumours, MRI voxels, tumour cords, and individual cells (Figure 1A). MRI voxels are 600 × 600 × 3000 *μ*m rectangular cuboids whose size is specified by spectrometer resolution. Because the average spacing between blood vessels in tumours is much less than these limiting dimensions, a set of assumptions were made about the tissue inside each voxel. These assumptions were necessary to interpret DCE-MRI transport information and couple the disparate length scales. The primary assumption was that the tissue within each voxel was uniform. Although this is a considerable simplification, it is the only way to connect MRI data to therapeutic predictions without specific information about the vasculature geometry within each voxel. To implement this assumption, we assumed each voxel to contain multiple identical repeating Krogh tumour vessel cords (Krogh, 1919a, 1919b), which are cylinders of tumour tissue surrounding a central blood vessel (Figure 1A). Longitudinal gradients and the space between cylinders were neglected. Neglecting this space has previously been shown to have minimal effects (Groebe, 1995). It was also assumed that there was no transport between voxels, which could not be observed because of the coarse temporal resolution of DCE-MRI. The computational model characterises tumour behaviour by summing the predictions obtained for individual component voxels (Figure 1A).

Two mechanisms affected tissue growth within voxels: (1) cell growth, which increased cord width; and (2) angiogenesis, which increased the number of cords (Figure 1B). The rates of cell growth and death were controlled by the delivery of nutrients and drugs from vessels into the interstitial space, as we have described previously (Venkatasubramanian *et al*, 2006, 2008). These rates were calculated by integrating the effects of nutrient and drug diffusion, cell-cycle progression, intracellular metabolism, cellular drug effects, and drug pharmacokinetics (Figure 1C; see Supplementary Information). Cells consumed three nutrients: glucose, oxygen, and lactate, whose uptake rates were dependant on local concentration and the stoichiometry of glycolysis and the TCA cycle (Venkatasubramanian *et al*, 2006). The time-varying concentration of paclitaxel in the vessel following i.v. administration was described by a three-compartment pharmacokinetic model (Gianni *et al*, 1995). The cytotoxicity of paclitaxel depended on the extracellular concentration and was assumed to be saturable (Venkatasubramanian *et al*, 2008). Uniform effective diffusion coefficients were assumed for all molecules. Spatial transport heterogeneity was attributed to variations in vascular permeability, which was determined by quantifying Gd-DTPA transport.

### Angiogenesis

Angiogenesis was modelled by increasing the number of cords in a voxel (Figure 1B). Because all cords in a voxel were assumed to be identical, it was not necessary to consider their arrangement, only the number per voxel, *ω*. Angiogenesis was controlled by the diffusion of angiogenic factors (assumed to be predominantly vascular endothelial growth factor, VEGF), produced by tumour cells in hypoxic microenvironments (Hockel and Vaupel, 2001). The rate of angiogenesis was dependant on three parameters: angiogenic sensitivity, *α*; the number of vessels, *ω*; and the concentration of VEGF at the vessel wall, *C*_VEGF_^vessel^. 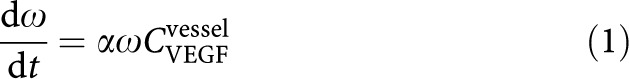


Angiogenic sensitivity, *α*, is a new parameter introduced here to capture spatial variations in cellular response to hypoxia and vessel response to VEGF. The production of VEGF was dependant on local oxygen concentration in the interstitial space, *C*_Ox_. 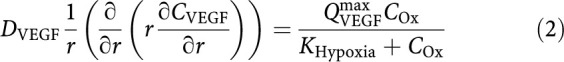


The diffusivity of VEGF, *D*_VEGF_; the maximum rate of VEGF formation, *Q*_VEGF_^max^; and the hypoxic sensitivity, *K*_Hypoxia_, were 1.13 × 10^−6^ cm^2^ s^−1^, 2.95 × 10^−17^ pmol *μ*m^−2^ s^−1^, and 10 mm Hg respectively (Hockel and Vaupel, 2001; Mac Gabhann and Popel, 2005; Mac Gabhann *et al*, 2006). The concentration of VEGF in the vessel wall was assumed to be proportional to the flux of VEGF across the vessel lining (see Supplementary Information).

### Tumour growth simulations

Overall tumour growth and drug response dynamics were determined by a four-step process (Figure 1D): (1) local transport parameters, *K*^trans^ and *f*_V_ (vascular volume fraction), were calculated for each voxel in a tumour as described below; (2) transport parameter pairs were converted into equivalent parameter pairs of angiogenic sensitivity, *α*, and average vessel permeability, *P*; (3) *α* and *P* parameter pairs were used to determine the local rate of cord growth, *μ*_G_, and the relative decrease in volume due to drug administration, Δ*V*; and (4) local rates were summed over all included voxels to obtain overall tumour values.

To streamline tumour analysis, we generated maps between parameter pairs. For each (*K*^trans^, *f*_V_) pair there existed one unique (*α*, *P*) pair and one unique (*μ*_G_, Δ*V*) pair. The implementation of parameter mapping rendered tumour analysis a rapid matrix multiplication and element summation process. Parameter maps were generated by simulating tumour cords with all physiological feasible values of *α* and *P* and determining the corresponding values of *K*^trans^, *f*_V_, *μ*_G_, and Δ*V*. The *α* and *P* grid consisted of 3600 points (60 on a side) spanning *α* values from 1 × 10^−5^ to 1 × 10^1^ mM per day and *P*-values from 1 × 10^−2^ to 1 × 10^2^ cm min^−1^.

To calculate tumour growth, *μ*_G_, and drug response, Δ*V*, we ran simulations for each initiating *α* and *P* pair. Transport parameters, *K*^trans^ and *f*_V_, were calculated from the final steady-state solutions. Simulations were initialised with a vessel surrounded by a thin layer of proliferating cells. Within each cord, volume increased and decreased as a function of radius due to cell growth and death as described above and in the Supplementary Information (Venkatasubramanian *et al*, 2006, 2008). The total rate of voxel volume change, d*V*/d*t*, was related to the rate of change in the number of cords, d*ω*/d*t*, and the rate of change of the cord thickness, d*R*_cord_/d*t*. 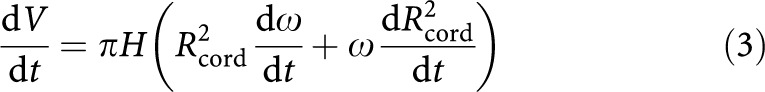


Here *H* is the arbitrary height of a voxel and is a parameter that had no effect on simulation results. Simulations were run until the rate of cord expansion, d*R*_cord_/d*t*, approached zero. This end point reflects the assumption that at the time of MRI acquisition vessel density in each voxel was not changing with time. Voxel vessel density was calculated from the final cord radius: *f*_V_=*R*_vessel_^2^/*R*_cord_^2^. The radius of all vessels, *R*_vessel_, was assumed to be constant throughout all tumours and equal to 20 *μ*m (Konerding *et al*, 2001).

The transvascular transport parameter, *K*^trans^, was determined by computationally introducing Gd-DTPA into each cord. The concentration in the blood plasma decreased according to the vascular input function, which was assumed to be an exponential function with a time constant of 6.94 (Tofts and Kermode, 1991). The commonly used second biexponential time constant (90.1 min) (Tofts and Kermode, 1991) did not affect Gd-DTPA concentration over the short investigated timescale. The rate of Gd-DTPA extravasation was controlled by the vascular permeability, *P*, and an effective tissue diffusivity of 2.6 × 10^−6^ cm^2^ s^−1^ (Thelwall *et al*, 2001). The total Gd-DTPA concentration profile was calculated for a 10 min period. This profile was subsequently used to calculate *K*^trans^ using the two-component method describe below (Figure 1D).

Overall tumour volume was determined by summing up the number of voxels within a tumour and multiplying by the volume of each voxel. For growth and drug simulations, the volume of each voxel was determined relative to its initial volume (0.6 mm × 0.6 mm × 3 mm). Tumour growth rate, *μ*_G_, was found by predicting tumour volume for 360 days at 60-day intervals and fitting the values to an exponential function. Tumour drug response was determined for each cord by introducing paclitaxel for eight biweekly cycles. This schedule is similar to that used at Baystate Medical Center for neoadjuvant therapy. The drug response, Δ*V*, was defined as the volume after treatment relative to the original volume.

### Local transport parameters

Two transport parameters were determined from each DCE-MRI data set using a two-compartment model (Figure 1D): the transvascular transport rate constant, *K*^trans^ and the vascular volume fraction, *f*_V_ (Harrer *et al*, 2004; Hylton, 2006; Martel, 2006; Zahra *et al*, 2007; Turnbull, 2009). To reduce signal noise, we pre-processed all images: individual voxel enhancements were averaged with eight neighbouring voxels. This smoothing reduced spatial resolution but enabled analysis of the intrinsically noisy images provided by the clinic. The concentration of Gd-DTPA in each voxel was assumed to be linearly proportional to the voxel signal enhancement, *E*, which was calculated by normalising the intensity, *I*, by the initial, unenhanced intensity, *I*_0_, *E*=(*I*−*I*_0_)/*I*_0_ (Chuthapisith *et al*, 2006; Lonning, 2007; Zahra *et al*, 2007). In this two-compartment model (see Supplementary Information) the sum of Gd-DTPA in the vascular and extra-vascular spaces contributed to the total measured signal enhancement (Figure 1D). Mass flux between the compartments was assumed to be linearly dependant on the concentration difference and the transvascular transport parameter, *K*^trans^ (Tofts, 1997; Harrer *et al*, 2004).

The signal enhancement profile of every voxel in every image was analysed individually using iterative optimisation. Values for the two adjustable parameters, *f*_V_ and *K*^trans^, were tuned until calculated enhancement profiles best matched measured profiles. Parameter estimation was performed using least-squares analysis and the Matlab optimisation routine *fmincon*. Tumour boundaries were defined as regions with *f*_V_ >2% or *K*^trans^ >0.005 min^−1^. All completely surrounded interior regions were included and disconnected regions 5 pixels and smaller were excluded. Methods for the construction of parameter maps and whole tumour images are described in Supplementary Information.

### Hypothetical homogeneous and heterogeneous tumours

The effect of heterogeneity was investigated by comparing predicted results for whole complex tumours to those with averaged transport properties. The behaviour of hypothetical homogeneous tumours was determined using a technique similar to that currently used clinically: a tumour signal enhancement was determined by averaging the signal enhancement of all voxels within a defined tumour boundary. The resulting signal enhancement time profile was analysed as described above to obtain average values for *K*^trans^ and *f*_V_. These values were subsequently converted into average growth rate and drug response.

To systematically test the effect of transport heterogeneity, we calculated growth rate and drug response for a set of hypothetical tumours with increasing *K*^trans^ variance. *K*^trans^ was assumed to have a *β*-distribution with a mean value of 0.015 min^−1^, which is the median value in the patient population. The relative variance was increased from 0.1 to 1.75 to include all values observed in the measured tumour set. The generation of hypothetical heterogeneous tumours is described in Supplementary Information. To quantitatively compare *K*^trans^ variance in the patient population, we normalized variances to eliminate the effect of different mean values. Variances were normalised by average *K*^trans^ values and responses were normalised by the growth rate and drug response of homogeneous tumours with the same mean.

### Statistical analysis

The existence of statistically significant correlations was determined by linearly regressing data and using one-factor ANOVA. A correlation was significant if the slope of the regression was significantly different from zero. Confidence intervals greater than 95% were considered significant.

## Results

### Cord growth and drug response parameters

**Figure 2:**
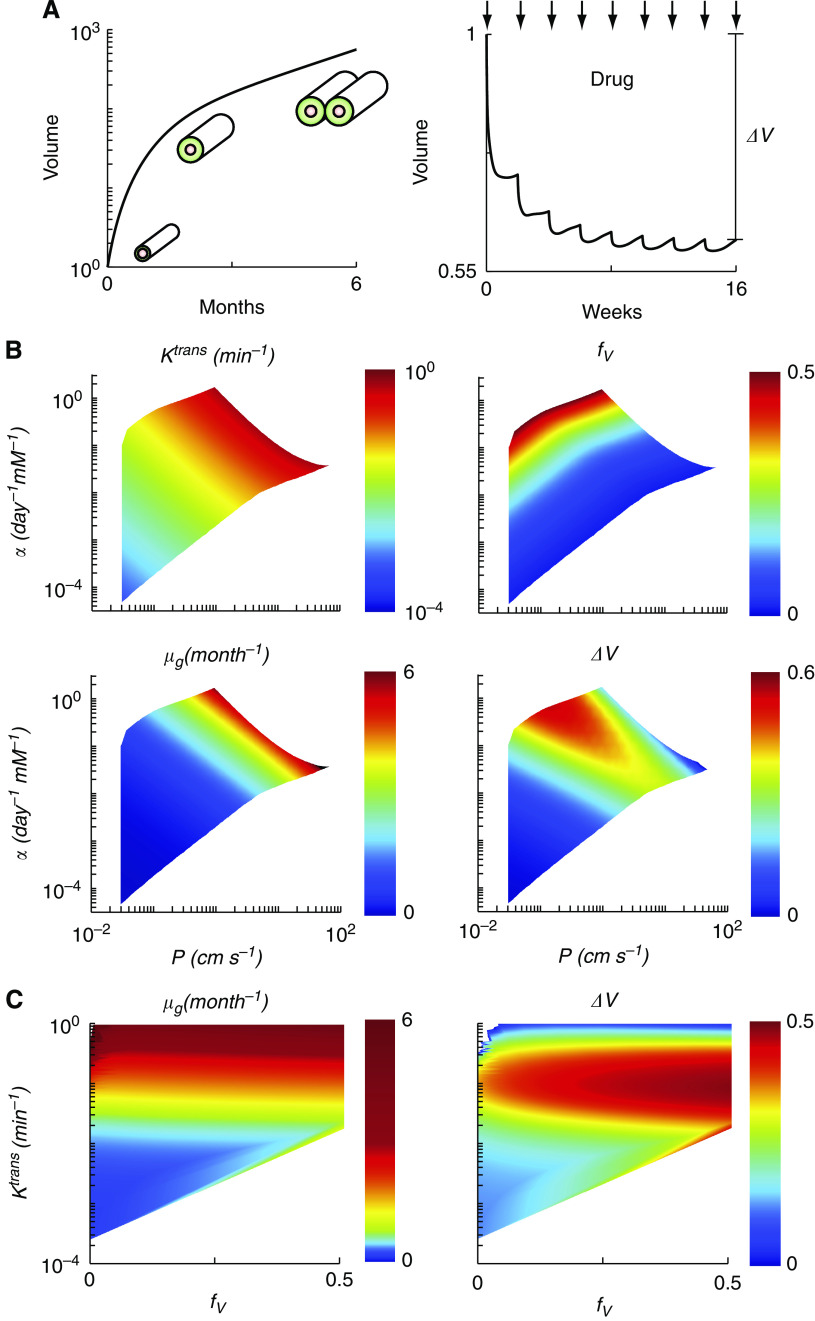
Tumour simulation results and parameter maps. (**A**) Representative growth and drug response from a single tumour simulation with parameters *α*=0.056 mM per day and *P*=2.51 cm min^−1^. Growth was characterised by an initial increase in cord radius followed by a period of increasing cord number due to angiogenesis. Response to eight biweekly paclitaxel administration cycles (arrows) was characterised by initial shrinkage followed by re-growth after drug washout. Drug response (Δ*V*) was defined as the relative difference between the initial and final volumes at the end of 16 weeks. (**B**) Parameter maps from *P* and *α* to *K*^trans^, *f*_V_, *μ*_G_ and Δ*V*. The units of *K*^trans^ and *μ*_G_ are min^−1^ and per month, respectively; *f*_V_ and *ΔV* are dimensionless. (**C**) Parameter maps directly from *K*^trans^ and *f*_V_ to *μ*_G_ and Δ*V*.

Growth rates and the drug responses of whole tumours were determined by summing the behaviour of individual tumour cords. Cord responses were determined over the entire range of observed input parameters, *α* and *P* (Figure 2). Simulation of the cord model with parameters *α*=0.056 mM per day and *P*=2.51 cm min^−1^ show characteristics that were observed for all cords (Figure 2A). Typically, cords grew in the absence of drug and shrank in the presence of drug (Figure 2A). Tumour cord growth was characterised by a fast exponential cell growth phase followed by a slower exponential angiogenic phase (Figure 2A). Initially the cord consisted of a single vessel surrounded by a thin layer of proliferating cells. At early times cells were well nourished and cord volume increased exponentially. As the cord grew, nutrient limitations appeared in the periphery causing growth cessation and cell death. Hypoxia in the periphery induced the production of angiogenic factors and caused new cords to form.

Tumour cord drug response was evaluated by simulating the administration of eight biweekly cycles of paclitaxel (Figure 2A). This schedule was designed to be similar to a typical administration pattern of neoadjuvant therapy. Each treatment with paclitaxel caused a reduction in volume followed by growth once drug had cleared from the blood (Figure 2A). Typically, the first response to drug was greatest and cord volume eventually stabilised with subsequent treatments (Figure 2A). The drug response was controlled by the combined effects of drug efficacy, drug transport limitations, cell growth, and angiogenesis. The overall effect of the 8-week drug cycle was characterised by the difference between initial and final volumes, Δ*V*.

Parameter maps were produced from model simulations to interpret the transport parameters extracted from MRI (Figure 2B). Maps were created between the inputs to the computational model (*α*, *P*), the transport parameters (*K*^trans^, *f*_V_), and the local responses (*μ*_G_, Δ*V*). Ultimately, direct maps between the directly measured transport parameters (*K*^trans^, *f*_V_) and local responses (*μ*_G_, Δ*V*) were created (Figure 2C), further simplifying analysis. This was possible because the relationships between the parameter pairs were one-to-one. White space on the maps represents regimes where cords shrank and mathematical solutions were not attainable. Parameter values in these regions were not observed in clinical tumours. Several anticipated relationships were identified in the parameter maps (Figure 2B). As expected, the overall transport coefficient, *K*^trans^, increased with increasing vessel permeability, *P*. Vessel density, *f*_V_, increased with increasing angiogenic sensitivity, *α*, and decreased with increasing permeability, *P*, because angiogenic sensitivity reduced cord saturation size and permeability increased nutrient supply to peripheral cells. Growth rate, *μ*_G_, increased with *P* and *α* because both increased nutrient availability.

Simulations of cord drug response, Δ*V*, generated two unexpected relationships. First, *f*_V_ had minimal effect on Δ*V* (Figure 2C). It was anticipated that a greater drug supply would have more effect on cord volume. The lack of response suggests that increased re-growth compensated for increased death. Second, the relationship between *K*^trans^ and Δ*V* was non-linear. At both low and high values of *K*^trans^ drug response was less (Figure 2C, right). The greatest drug response was observed at moderate values of *K*^trans^ (Figure 2C, right).

### Whole tumour analysis

**Figure 3:**
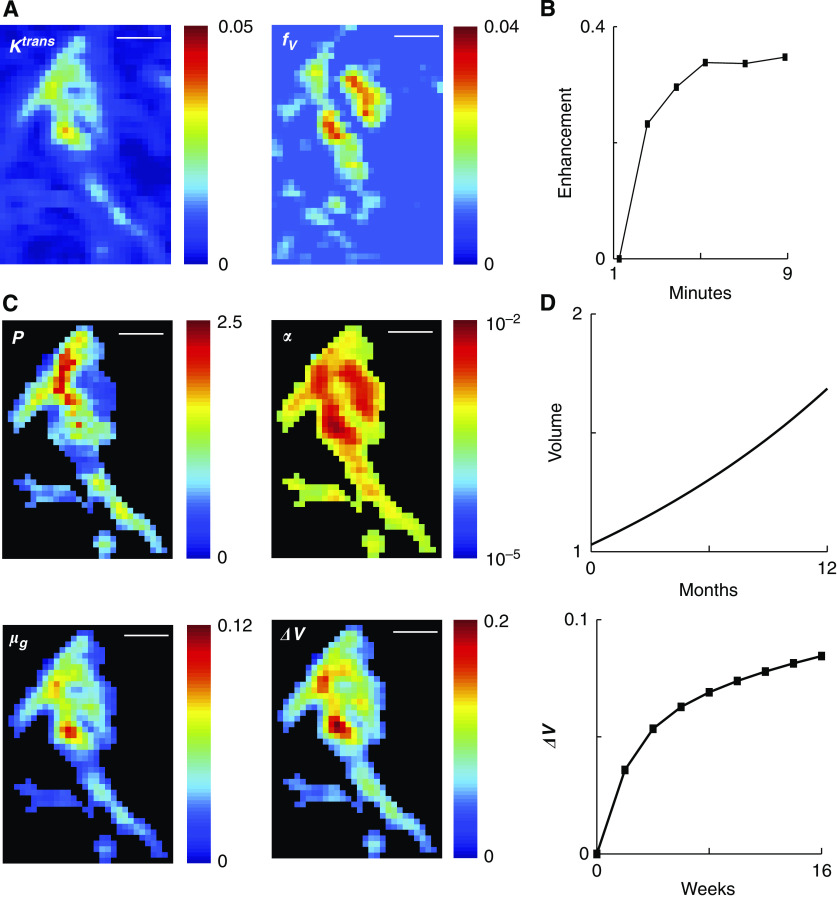
Whole tumour predictions for growth and drug response based on local DCE-MRI data. (**A**) Images of local *K*^trans^ and *f*_V_ values for a representative MRI slice from patient 2. The units of *K*^trans^ are min^−1^ and *f*_V_ is dimensionless. Scale bar is 5 mm. (**B**) Representative DCE-MRI signal intensity profile for one voxel within the tumour of patient 2. (**C**) Images of local *P*, *α*, *μ*_G_, and Δ*V* values for the representative slice in (**B**). Colour scales are linear for *P*, *μ*_G_, and Δ*V* and log scale for *α*. The units of *P*, *α*, and *μ*_G_ are cm min^−1^, mM per day, and per month, respectively; and Δ*V* is dimensionless. Scale bar is 5 mm. (**D**) Tumour growth and drug response predictions for patient 2 obtained by summing the behaviour of all voxels in all slices comprising the tumour.

**Figure 4:**
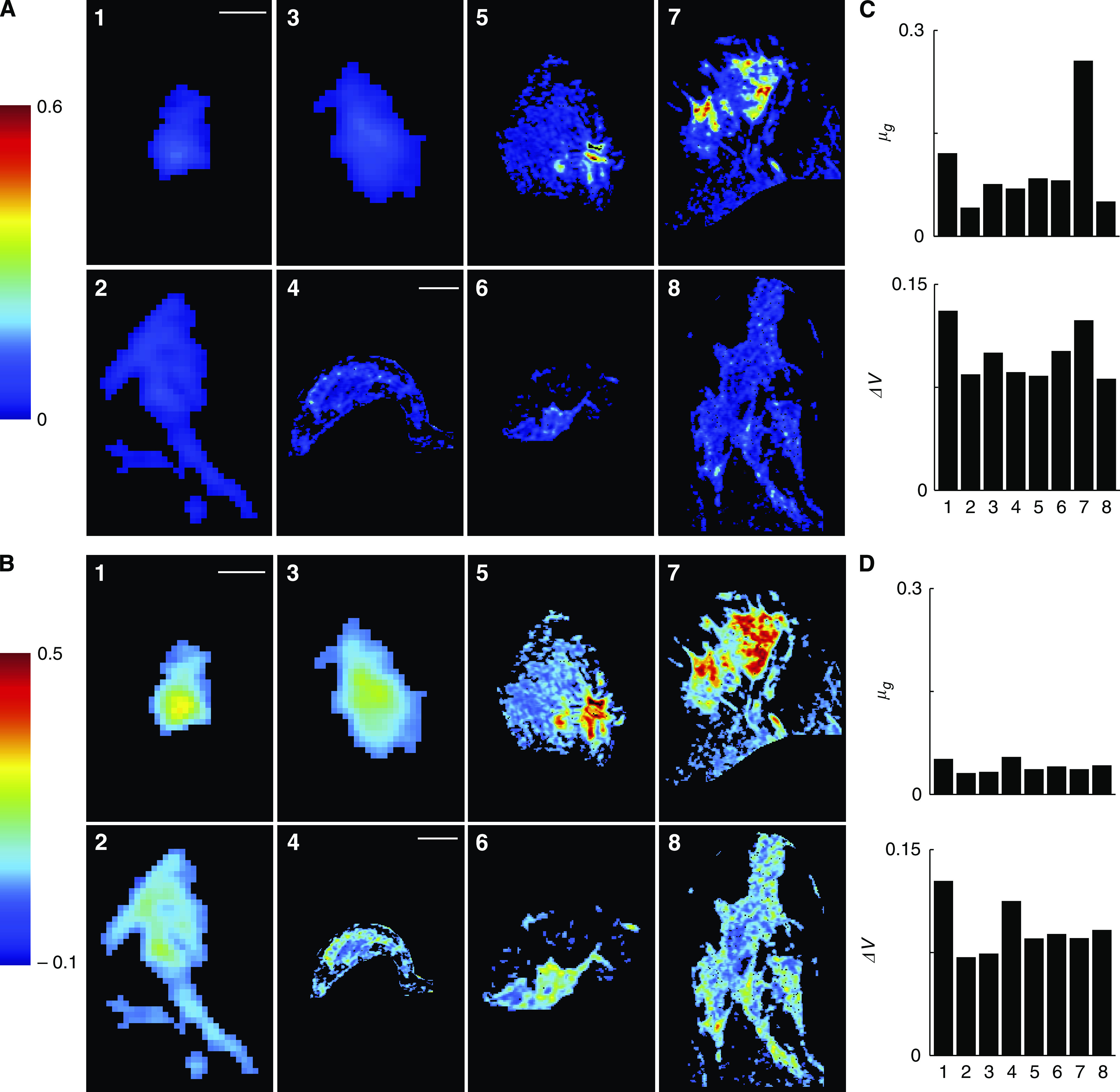
Heterogeneity of growth rate and drug response calculated for eight patients. (**A**, **B**). Representative slices showing local values of *μ*_G_ (**A**) and Δ*V* (**B**) for eight patients arranged in order of increasing size. Regions outside the identified tumour are blackened. Scale bar is 5 mm for patients 1–3 and 2 cm for patients 4–8. (**C**) Growth rate (*μ*_G_) and drug response (Δ*V*) predictions were determined by summing local behaviours in (**A**) and (**B**) for all slices in each tumour. (**D**) Predictions for homogeneous tumours with single *K*^trans^ and *f*_V_ values calculated from signal intensity profiles averaged across whole tumours.

Tumour growth rates and drug responses were calculated from DCE-MR images for the eight patients in the study (Figures 3 and 4). For each tumour, multiple slices were acquired at five times after Gd-DTPA administration. The images were processed to generate signal enhancement profiles for each tumour voxel (Figure 3B). These profiles were used to estimate local values for the transport rate, *K*^trans^, and vascular volume fraction, *f*_V_ (Figure 3A). Every observed tumour contained voxels with a wide distribution of vasculature and transport values. Although the use of a fixed AIF can lead to systematic errors (Tofts and Kermode, 1991; Buckley *et al*, 2004), the measured values for *K*^trans^ and *f*_V_ were consistent with previously reported values (Harrer *et al*, 2004). Regions of high transport did not necessarily correlate with regions of high vessel density, which is also consistent with previous results (Gillies *et al*, 2002). Tumour boundaries were determined from the union of the transport and vessel density images as described in Materials and Methods. The local transport parameters (Figure 3A) and the parameter maps (Figure 2B and C) were used to calculate unique local values for *α*, *P*, *μ*_G_, and *ΔV* (Figure 3C). By summing the behaviour of all voxels, we predicted the growth rate of the entire tumour and the overall response to eight cycles of drug administration (Figure 3D).

This analysis procedure was repeated for all eight patients in the study (Figure 4). Generally, regions of rapid growth also had the highest response to drug treatment (Figure 4A and B). Summing the behaviour across all voxels predicted the response of whole tumours (Figure 4C). Average growth rates and drug responses were in the ranges 0.06–0.3 per month and 8–13% respectively. When organised in order of increasing tumour size, neither growth rate nor drug response correlated with size (Figure 4C and D).

To investigate how heterogeneity affected predicted outcomes, we used average signal enhancement profiles to simulate homogeneous tumour behaviour (Figure 4D). Averaging the signal enhancement underestimated growth rate and inaccurately predicted drug response trends. The predicted growth rates for the homogenous tumours were in the range 0.03–0.06 per month, which was ∼20% of values obtained from full voxel-by-voxel analysis. For some tumours, homogeneous analysis considerably underestimated drug response (patients 2, 3, 6, and 7) and for others it considerably overestimated response (patients 4 and 8).

### Correlations between parameters and outcomes

**Figure 5:**
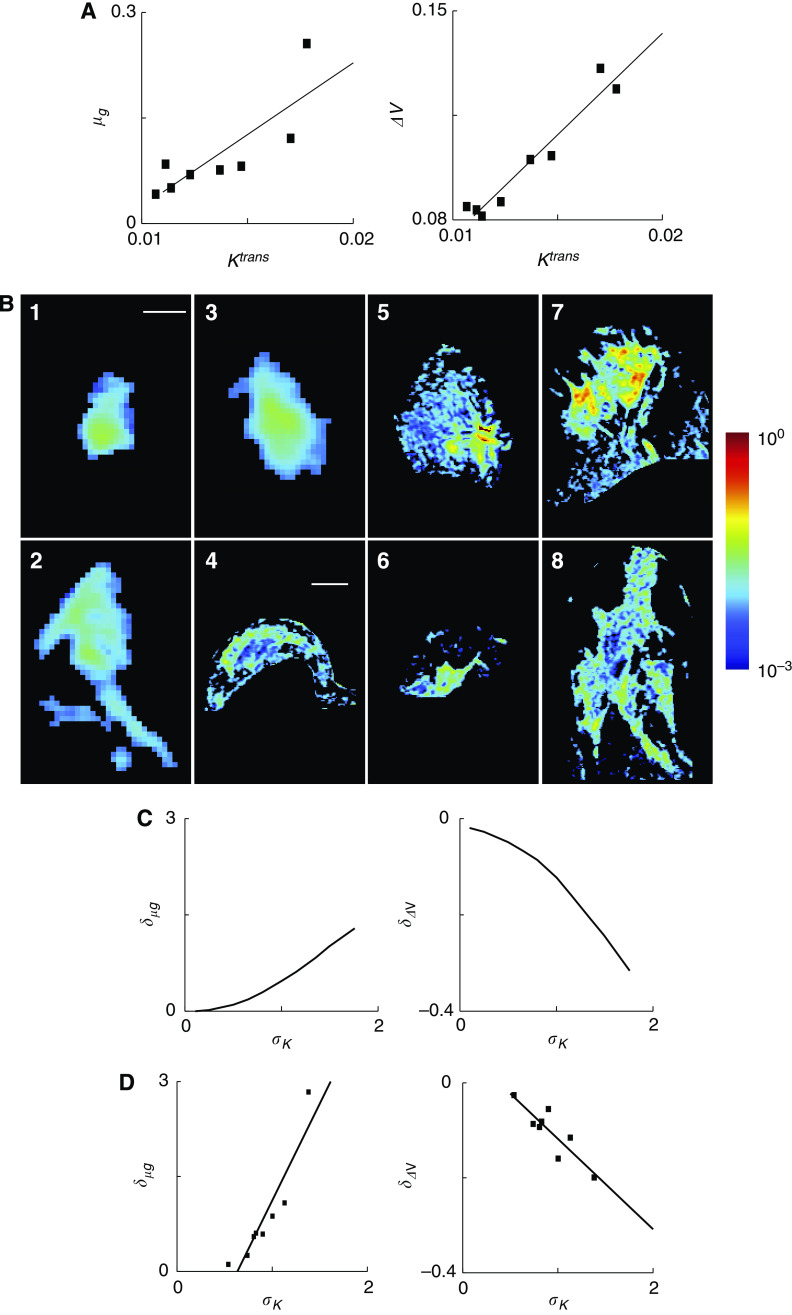
*K*^trans^ and tumour heterogeneity correlate with growth rate and drug response predictions. (**A**) Overall growth rate and drug response were correlated with average *K*^trans^ values across the patient population. Slopes of linear correlation functions were both significantly greater than zero (*P*<0.05). (**B**) Representative slices showing local *K*^trans^ values. Regions outside the tumour are blackened. Scale bars is 5 mm for patients 1–3 and 2 cm for patients 4–8. (**C**) Increasing the standard distribution of *K*^trans^ (*σ*_*K*_) in a hypothetical heterogeneous tumour increased relative growth rate (*δ*_*μ*G_) and decreased relative drug response (*δ*_ΔV_). Relative growth rate is defined as the difference in growth between the heterogeneous tumour and a hypothetical homogeneous tumour with the same average *K*^trans^ value. Relative drug response is defined similarly. (**D**) For the eight patients, an increasing standard distribution of *K*^trans^ (*σ*_*K*_) correlated with increased relative growth rate (*δ*_*μ*G_) and decreased relative drug response (*δ*_ΔV_; *P*<0.05).

Of the four input parameters (*K*^trans^, *f*_V_, *α*, and *P*) the transport parameter, *K*^trans^, had the strongest correlation with the predictive responses (*μ*_G_ and Δ*V*; Figure 5). In the patient population, simulations predicted that tumours with higher average *K*^trans^ values would have significantly (*P*<0.05) higher growth rates and better responses to chemotherapy (Figure 5A). The correlation coefficients between *K*^trans^ and *μ*_G_ and Δ*V* were 0.82 and 0.97 respectively. This trend was also observed on the voxel scale; tumour regions with high *K*^trans^ values (Figure 5B) also had high growth rates and drug responses (Figure 4A and B). The global effect of the input parameters on responses was further quantified by analysing hypothetical homogeneous tumours. These hypothetical tumours had single values of *K*^trans^ and *f*_V_ within the range of values measured in the patient population (Figure 5A; Supplementary Figure S1). The coefficients of determination between the parameters *K*^trans^, *P*, *f*_V_, and *α* and the drug response, Δ*V*, were 0.90, 0.57, 0.096 and 0.04 respectively, indicating that *K*^trans^ is considerably more predictive than the other three parameters (see Supplementary Information).

### Effect of heterogeneity on prediction of drug response

Heterogeneity significantly increased tumour growth rate and decreased drug response (Figure 5C and D). Because it was the predominant parameter, tumour heterogeneity was characterised by the standard deviation of *K*^trans^, *σ*_*K*_. The relative standard deviation of *K*^trans^ in the patient population ranged from approximately 0.5 to 1.5 (Supplementary Figure S1). To analyse the effect of heterogeneity a set of hypothetical tumours was created with constant mean *K*^trans^ and *f*_V_ values and a range of values for the standard deviation of *K*^trans^. In this hypothetical set, increasing the standard deviation of *K*^trans^ increased growth rate and decreased drug response (Figure 5C). An identical pattern was observed in the patient population: increasing the standard deviation of *K*^trans^ significantly increased growth rate (*P*<0.05) and decreased drug response (*P*<0.05; Figure 5D). The correlation coefficients between *σ*_*K*_ and *δ*_*μ*G_ and *δ*_ΔV_ were 0.88 and −0.92 respectively. The mean *K*^trans^ value and the standard deviation of *K*^trans^ had similar effects on growth rate but opposite effects on drug response. Increasing the mean and standard deviation both increased growth rate. However, increasing the mean improved response and increasing the standard deviation decreased response.

## Discussion

Here we describe a predictive multi-scale tumour model that incorporates drug transport, angiogenesis, pharmacokinetics, and intracellular metabolism. A method was developed to rapidly determine transport parameters from individual patient DCE-MRI acquired in a clinical setting. Angiogenesis was modelled by extending the Krogh model to include formation of new blood vessels. The model framework introduced a new parameter, *α*, to describe the sensitivity of cells and vessels to different levels of oxygen and VEGF. The inclusion of this angiogenic sensitivity accounted for variations in vessel densities observed across tumours. Without its inclusion all tumour cords would have saturated at the same external radius. The model framework also predicts numerous testable tumour characteristics, including local oxygen and glucose concentrations, rates of cell proliferation, and the extent of necrosis. These predictions are useful because they can be tested (by histology or PET, for example) and because they begin to provide a mechanistic connection between imaging data and drug efficacy.

Because the mathematical model describes general physiological mechanisms, it has the potential to be generalisable to other treatments and tumour sites. Angiogenesis, intracellular metabolism, and tissue growth are aspects common to all solid tumours. Generalisability is supported by recent results showing that in a different site, glioblastoma, *K*^trans^ strongly correlates with overall survival and *K*^trans^ heterogeneity can distinguish between responding and non-responding patients (Sorensen *et al*, 2009). Complete validation of the model's generalisability will require further clinical investigations.

### Predominance of *K*^trans^

Of the parameters used in the model framework to characterise tumour behaviour, the local trans-vascular transport rate, *K*^trans^, had the greatest effect on tumour growth and drug response. Simulations showed that responses were more dependant on *K*^trans^ than vascular density, *f*_V_ (Figure 2C, left), vascular permeability, *P*, or angiogenic sensitivity, *α*. The predominance of *K*^trans^ can be also seen in the banding pattern of the parameter maps. In the maps from *α* and *P* to *K*^trans^, *μ*_G_, and Δ*V* (Figure 2B) similar bands run from the upper left to the lower right. These patterns indicate that *K*^trans^ captures a combined effect of permeability and angiogenic sensitivity that better predicts *μ*_G_ and Δ*V* than either parameter alone.

**Figure 6:**
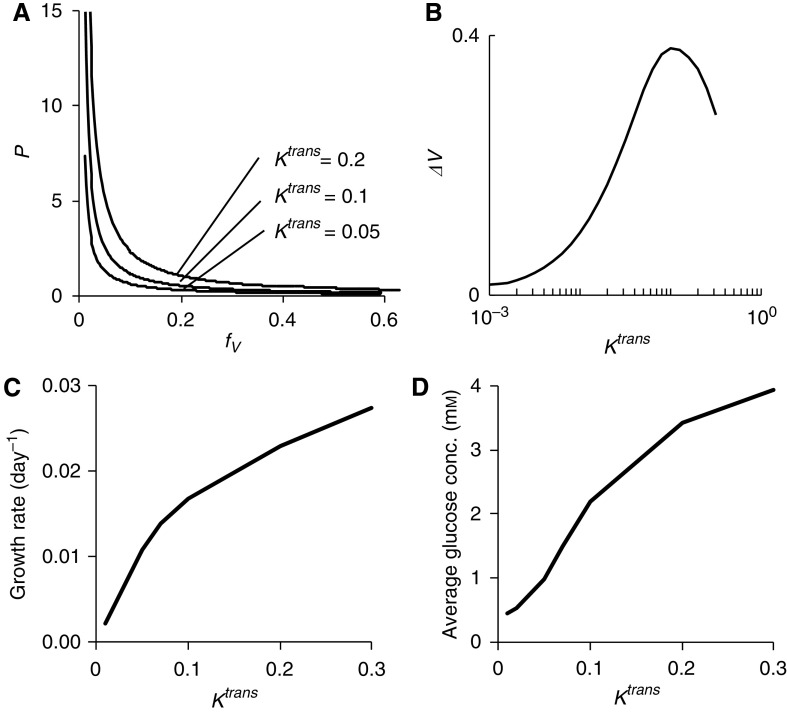
Physical characteristics of tumour cords as predicted by simulation. (**A**) At any given *K*^trans^ value, permeability and vessel density were inversely related. Increasing *K*^trans^ increased both *P* and *f*_V_. (**B**) The relationship between *K*^trans^ and Δ*V* (shown at *f*_V_=0.05) possessed an optimal drug response. (**C**, **D**) The cord growth rate (**C**) and the average cord glucose concentration (**D**) were both strongly dependant on *K*^trans^. Relations are shown at *f*_V_=0.05.

The physiological nature of the model enabled a mechanistic explanation of these relations between parameters (Figure 6). The connection between tumour growth (*μ*_G_) and *K*^trans^ (Figure 2C, left) was a consequence of the complex dependence of *K*^trans^ on vessel density, *f*_V_, and permeability, *P* (Figure 6A). Cords with high *K*^trans^ values transported more material into tumour tissue because of either higher vessel density (high *f*_V_) or greater leakiness (high *P*). At any given *K*^trans^ value, high vessel density was associated with low permeability, and low density was associated with high permeability (Figure 6A). When translated into tumour growth, poor delivery due to low vessel density was compensated by high permeability, resulting in a similar growth rate to cords with high vessel density and low permeability (Figure 2C, left). However, higher values of *K*^trans^ represented cords with both greater density and permeability (Figure 6A), which therefore received more nutrients and grew faster (Figure 2C, left).

The relation between *K*^trans^ and drug response was more complicated than tumour growth. The effect of *K*^trans^ on Δ*V* was non-linear and showed a maximum value for which there was an optimal drug response (Figure 2C, right and Figure 6B). This optimum was caused by the interaction of two competing factors observed in the cord simulations: (1) shrinkage in the presence of drug and (2) re-growth after drug washout. At low values of *K*^trans^, few cells were proliferating (Figure 6C) and were not responsive to drug. At high values of *K*^trans^, tumour shrinkage was limited because drug-induced cell death was outpaced by tissue re-growth, fueled by abundant nutrients (Figure 6D).

### Heterogeneity

Model simulations suggest that monitoring average transport behaviour is insufficient and that it is important to quantify tumour heterogeneity to predict drug response. Heterogeneity is important for two reasons. First, including only the average transport rate in our simulations poorly predicted the tumour drug response (Figure 4). These simulation results suggest that determining both the average and standard deviation of *K*^trans^ is necessary to predict drug response accurately. Second, the mean value of *K*^trans^ had a mixed effect but the variance was doubly detrimental. Tumours with high average *K*^trans^ values had fast growth rates but increased drug responses (Figure 5A). Highly heterogeneous tumours, however, grew fast and responded poorly to therapy (Figure 5C and D).

These seemingly contradictory effects were caused by different mechanisms. Heterogeneous tumours had higher growth rates because more regions were well supplied with nutrients. The model predicted that these regions grew more than an order of magnitude faster than regions with a nominal supply of nutrients (Figure 6C and D), and had a dominant effect on the overall tumour growth rate. Heterogeneous tumours had poor drug response because a large fraction of the tumours were not proliferating and were not responsive to drug (Figure 6C). The increase in volume reduction seen in regions with higher drug concentrations was small and did not compensate for the minimal response in large non-responsive regions.

## Conclusion

We have developed a model framework that integrates individual patient data into a tumour growth model, which accounts for intracellular metabolism, nutrient and drug diffusion, trans-vascular permeability, and angiogenesis. To the best of our knowledge this is the first report on the acquisition of transport parameters from individual patient DCE-MRI and their use in a predictive computational model. Simulations with this model suggested that *K*^trans^ is correlated with tumour aggressiveness because increased vessel growth and permeability provide more nutrients for cell proliferation. Simulations also suggested that tumour heterogeneity is a critical parameter to monitor because of its association with increased tumour growth and poor drug response. In addition the model simulations produced multiple testable hypotheses. For example the simulations suggested that (1) vessel density minimally affects tissue growth and drug response, (2) nutrient availability drives tissue growth, and (3) angiogenic rates vary across tumours. All the results suggest that DCE-MR images contain critical information that can predict therapeutic efficacy and that mathematically modelling has the potential to improve treatment decisions. We believe that this model is a first step towards the creation of a comprehensive computational method that will provide individual therapeutic predictions based on imaging data. Continued development of these methods has the potential to provide individualised patient predictions and improve overall cancer care.
