# Supplementary material for: Mechanistic modelling of dynamic MRI data predicts that tumour heterogeneity decreases therapeutic response
Source: Br J Cancer. 2010 Jul 13;103(4):486–97. doi: 10.1038/sj.bjc.6605773 (PMC2939778; doi:10.1038/sj.bjc.6605773)
Supplement: Supplementary Information [file 6605773x1.doc]

**Supplemental Information**

**Mechanistic Modeling of Dynamic MRI Data Predicts that Tumor Heterogeneity Decreases Therapeutic Response**

Raja Venkatasubramanian, Richard B. Arenas, Michael A. Henson, Neil S. Forbes

***Model Structure***

***The model formulation is an extension of our previous models that incorporated cell-cycle transition, drug transport, drug pharmacodynamics and drug pharmacokinetics (Venkatasubramanian et al, 2006; Venkatasubramanian et al, 2008). The model consists of cell balance equations and mass balance equations for nutrients and drugs.***

| $\frac{\partial n_{i}}{\partial t}+\nabla\cdot(\nu n_{i})=g_{i}$ | $\left.\frac{\partial n_{i}}{\partial r}\right\vert_{ r=R_{cord}}=0$ | (S1) |
| --- | --- | --- |
| $\nabla\cdot(\nu)=\varphi_{generation}^{volume}-\varphi_{loss}^{volume}$ | $\left.v\right\vert_{ r=R_{vessel}}=0$ | (S2) |
| $\frac{\partial C_{j}}{\partial t}+D_{j}\Delta^{2}C_{j}+\nabla.(\nu C_{j})=0$ | $\left.\frac{\partial C_{j}}{\partial r}\right\vert_{r=R_{cord}}=0 \left.D_{j}\frac{\partial C_{j}}{\partial r}\right\vert_{r=R_{vessel}}=P(C_{j}^{lumen}-C_{j}^{vessel})$ | (S3) |
| $D_{k}\Delta^{2}C_{k}=Q_{k}$ | $\left.\frac{\partial C_{k}}{\partial r}\right\vert_{r=R_{cord}}=0 \left.D_{k}\frac{\partial C_{k}}{\partial r}\right\vert_{r=R_{vessel}}=P(C_{k}^{lumen}-C_{k}^{vessel})$ | (S4) |

Equation (S1) is a balance equation representing conservation of cell number (*n_i_*) for *i-*type cells (proliferating, p; quiescent, q; or dead, d). The left hand side of the equation consists of an accumulation term followed by a convective term that accounts for the cell convection caused by the expansion/contraction of tumor tissue. The right hand side (*g_i_*) accounts for changes in the number of cells in each phase due to transitions between the phases and cell death. Gradients in convective cell velocities (*v*, Eq. S2) are created by volume changes during cell growth ($\varphi_{generation}^{volume}$) and death ($\varphi_{loss}^{volume}$).

Drug accumulation was balanced by diffusive and convective transport (Eq. S3), where *C_j_* is the extracellular drug concentration and *D_j_* is the effective drug diffusivity in tumor tissue. The time scales for drug transport and tumor growth were assumed to be similar, and drug uptake by cells was assumed to be negligible. In the nutrient balance (Eq. S4) diffusion was assumed to be balanced by consumption. The variables *C_k_*, *D_k_* and *Q_k_* are local concentration, diffusion coefficient, and uptake rate of nutrient *k*, respectively. Consumption of three nutrients: glucose, oxygen and lactate were assumed to be necessary for cell growth. Because the molecular weights of the nutrients are small, it was assumed that transport was fast compared to tumor growth and that nutrients do not accumulate. The nutrient balance is similar to the VEGF balance described in the text (Eq. 2), with the exception that VEGF was produced and nutrients were consumed. The pseudo-steady state assumption was also applied to VEGF.

For all balances, a Neumann-type symmetry condition was imposed at the cord edge. This constraint was implemented as a zero-gradient boundary condition for the cell, nutrient and drug balances (Eqs. S1, S3, and S4). The velocity was assumed to be zero at the vessel wall (Eq. S2). The second boundary condition for the nutrient and drug balances (Eqs. S3 and S4), assumes that the flux at the vessel wall was dependent on the permeability, *P*, and the difference between the tissue, *C^vessel^*, and blood lumen, *C^lumen^*, concentrations.

Two major changes were made in the model structure from the model described previously (Venkatasubramani*an et* al, 2006; Venkatasubramani*an et* al, 2008). First, all equations were formulated in cylindrical coordinates with blood as the nutrient source at the center of each tumor vessel cord. A no-flux boundary condition was enforced at the exterior to preserve symmetry between adjacent cords. The Dirichlet-type boundary condition at the vessel-tumor cell interface was modified to a Robin-type boundary condition to incorporate vessel wall permeability. The lumen oxygen concentration was set to 0.05 mM, which is the average oxygen concentration in blood plasma (William D. McArdle, 2006).

Second, the number of cell types was reduced to two live phases: proliferating and quiescent. We have shown previously that tracking all cell cycle phases has minimal effect on drug response predictions (Venkatasubramani*an et* al, 2008). Proliferating cells were assumed to double every 30 hrs and the time scale of proliferating cells entering quiescence was increase proportionally to 41.67 hrs. The critical ATP threshold for cell cycle arrest of proliferating cells was assumed to be the same as that of G1 cells in the previous model (Venkatasubramani*an et* al, 2008). Cell balances (Eq. S1) described the number density of cells in each phase as a function of radial position and time. The transition rates between the phases are described by kinetic expressions dependent on local ATP generation rates (Eq. S5).

| $\begin{matrix} g_{P}=\left(2\mu_{P}^{P}n_{P}+\mu_{P}^{Q}n_{Q}\right)-\left(\hat{\mu}_{D}^{P}n_{P}+\hat{\mu}_{Q}^{P}n_{P}\right) \\ g_{Q}=\hat{\mu}_{Q}^{P}n_{P}-\left(\mu_{P}^{Q}n_{Q}+\hat{\mu}_{D}^{Q}n_{Q}\right) \end{matrix}$ | (S5) |
| --- | --- |

The transition rates between the phases depend on the ATP generation rates.

|  | (S6) |
| --- | --- |

The effect of drug on cell death was dependant on the extra-cellular concentration, and was assumed to be saturable at high concentrations.

| $\mu_{D}^{drug}=\frac{\mu_{D,max}^{drug}C_{drug}}{K_{D}^{drug}+C_{drug}}$ | (S7) |
| --- | --- |

***Concentration of VEGF at the vessel wall***

The concentration of VEGF at the vessel wall was assumed to be proportional to the flux of VEGF across the vessel lining. This assumption was based on the following relations. By mass balance, the total flux of VEGF out of surrounding tumor tissue is equal to the flux of VEGF across the vessel lining.

| $\left.D_{VEGF}\frac{\partial C_{VEGF}}{\partial r}\right\vert_{r=R_{vessel}}=k\left(C_{VEGF}^{vessel}-C_{VEGF}^{lumen}\right)$ | (S8) |
| --- | --- |

Here $C_{VEGF}^{lumen}$is the VEGF concentration in the vessel lumen and *k* is a lumped mass transfer coefficient that couples vessel permeability and radial mass transfer in the blood. At nominal blood velocities, $C_{VEGF}^{lumen}$approaches zero, resulting in a linear relationship between vessel wall concentration and VEGF flux.

| $\left.C_{VEGF}^{vessel}=\left(\frac{D_{VEGF}}{k}\right)\frac{\partial C_{VEGF}}{\partial r}\right\vert_{r=R_{vessel}}$ | (S9) |
| --- | --- |

The constant *D_VEGF_/k* was assumed to be a component of *α* (see equation 1) eliminating the need to explicitly calculate $C_{VEGF}^{lumen}$. The flux of VEGF across the vessel lining was found by solving Equation 2.

***Gd-DTPA pharmacokinetic model***

A two compartment model was used to describe Gd-DTPA pharmacokinetics. The two compartments were vascular and extravascular space (See Figure 1D). This model assumed uniform volume ratio of cells and extracellular matrix in the extravascular space. There is a bidirectional exchange of the contrast agent between vascular blood plasma and extravascular volume. The mass flux is assumed to be linearly dependent on concentration difference between the two compartments.

| $\frac{dC_{ev}}{dt}=K^{trans}(C_{v}-C_{ev})$ | (S10) |
| --- | --- |

where, *K^trans^* is the transvascular transport rate constant, and *C_v_* and *C_ev_* are the concentrations of Gd-DTPA in the vascular and extravascular spaces respectively. The concentration of Gd-DTPA in both spaces contributed to the total signal intensity measured in each MRI voxel.

| $C_{total}=f_{ev}C_{ev}+f_{v}C_{v}$ | (S11) |
| --- | --- |

where, *C_total_* is the average concentration measured in each voxel, and *f_ev_* and *f_v_* are the extravascular and vascular volume fractions. Because the two volume fractions were assumed to sum to one, only two independent parameters were needs to describe the transport properties of each voxel, *K^trans^* and *f_v_*.

| $f_{v}+f_{ev}=1$ | (S12) |
| --- | --- |

***Construction of parameter maps and whole tumor images***

Parameter maps were generated from non-uniform grid points by defining triangles using inbuilt *Matlab* function ‘*delaunay*’ such that no data points are contained in any triangle's circumscribed circle. The *Matlab* function ‘*trisurf’* was used to display the set of identified triangles as a surface. Tumor images of growth rate (*μ_g_*), drug response (*ΔV*), permeability (*P*) and angiogenic sensitivity (*α*) were determined by 2D interpolation for each voxel. For each combination of vascular volume fraction (*f_v_*) and local trans-vascular transport (*K^trans^*), unique values for *μ_g_*, *ΔV*, *P* and *α* were obtained from parameter maps. For the non-uniform set of gridpoints a set of triangles is determined using inbuilt Matlab® function ‘***delaunay***’ such that no data points are contained in any triangle's circumscribed circle. The Matlab function ‘***tsearch***’ is used to determined the enclosing delaunay triangle and a linear 2-D surface interpolation using the vertices of the identified triangle determines the relevant parameter.

***Generation of hypothetical heterogeneous tumors***


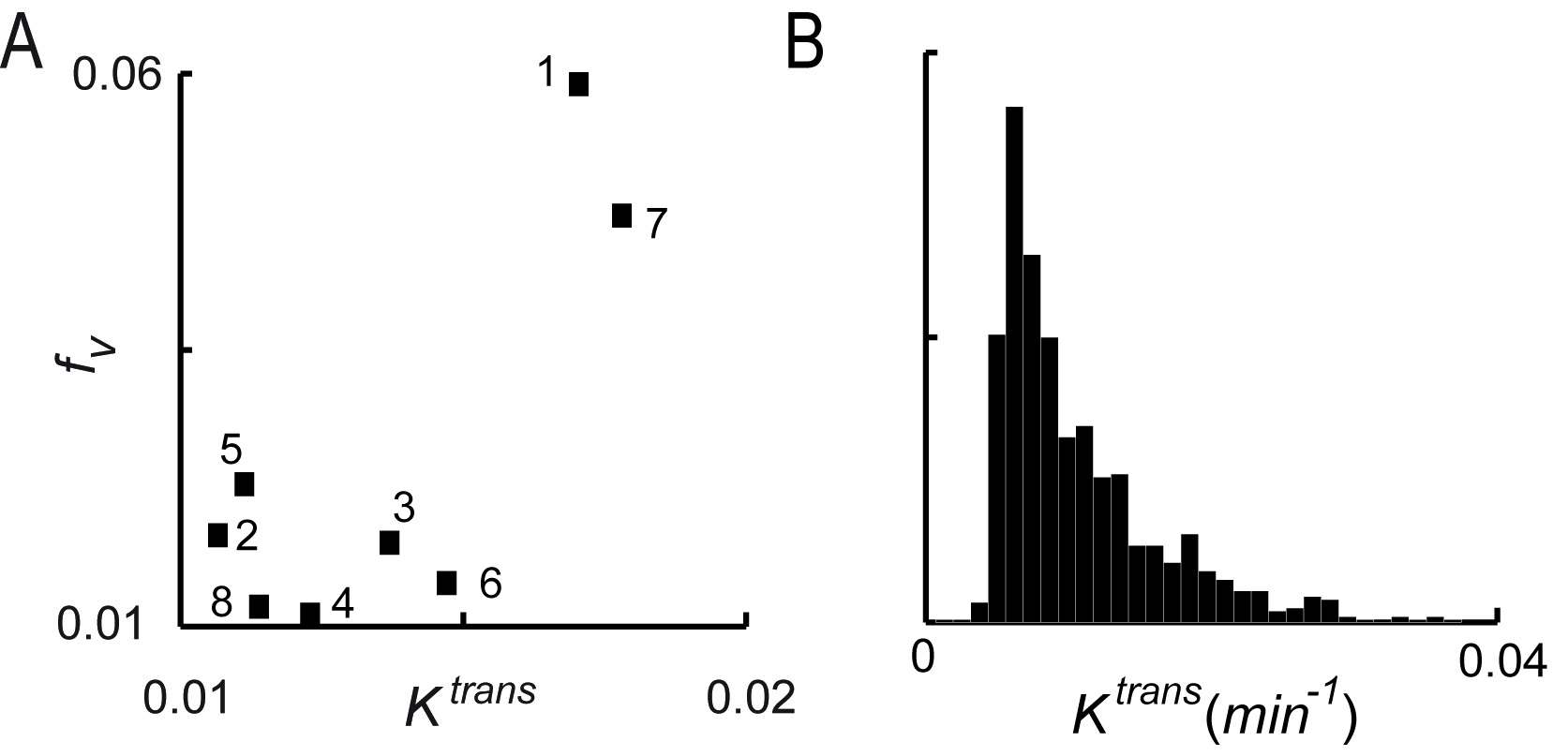


**Figure S1 Parameter distributions in patient population.** (A) Distribution of average *f_v_* and *K^trans^* values, annotated by patient number. (B) Distribution of *K^trans^* values in the tumor of patient 2.

Hypothetical heterogeneous tumors were formed with random distributions of transvascular transport (*K^trans^*) and vascular volume fraction (*f_v_*). *K^trans^* was assumed to be distributed according to beta distribution because of the high density of low values and long tail (Figure S1B). It was desired to use a distribution that only produced only positive values and had a limited range. Patient *K^trans^* populations were fit to four different distribution models: normal, Poisson, log normal, and beta distributions. Least squared error analysis showed that the beta distribution best fit the data for most of the patients.

To create hypothetical heterogeneous tumors, *K^trans^* was assumed to be distributed between 0 and 0.3 min^-1^ to match the values seen in the patient population (Figure S1A). Ten different hypothetical tumors were created with increasing variances and identical means. This method separated the effects of heterogeneity from the effects of *K^trans^*. Each tumor contained 60,000 voxels with independent *K^trans^* values. This large size ensured that the mean across all the tumors remained the same. A uniform distribution was used for the vascular volume fraction (*f_v_*) over a range from 0.01 – 0.21%. All hypothetical tumors had the same random sequence with uniform distribution for the vascular volume fraction.


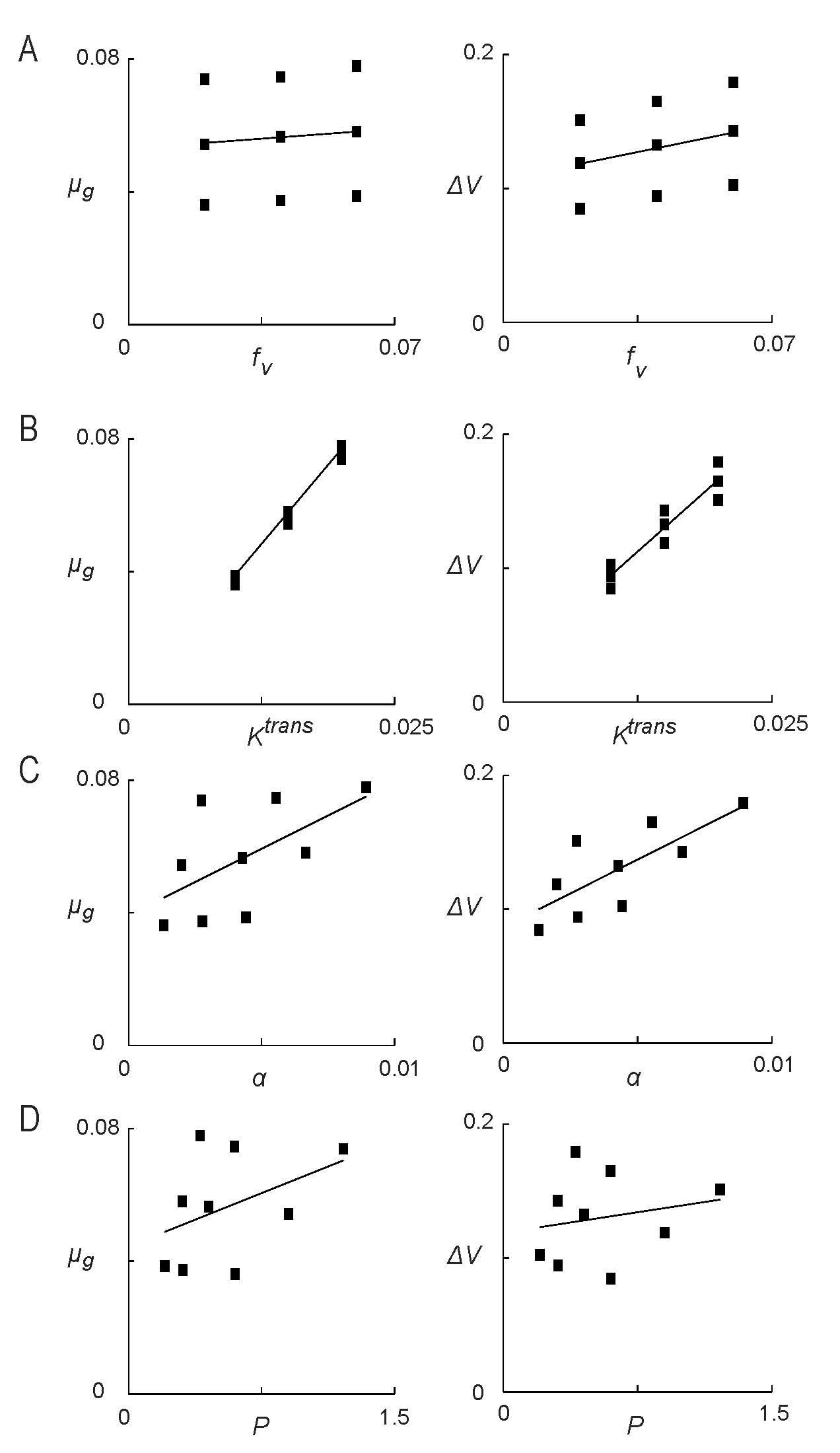


**Figure S2 Correlations between the input parameters (*K^trans^*, *f_v_*, *α*, and *P*) and the predicted outcomes (*μ_G_* and *ΔV*)**

***Correlations between parameters and outcomes***

The global effect of the input parameters on the responses was determined by analyzing nine hypothetical homogeneous tumors created in the range of parameters observed in the patient population: 0.1 to 0.2 for *K^trans^* and 0.02 to 0.06 for *f_v_* (Figure S1A). Over this range, growth rates and drug responses were more strongly dependant on the *K^trans^* than *f_v_* , *α*, or *P* (Figure S2). From these simulations results with hypothetical tumors coefficients of determination (Figure S1) between the input parameters and drug response, *ΔV*, were found to be 0.90, 0.57, 0.096 and 0.041 for *K^trans^*, *P*,  *f_v_*, and *α*, respectively. These simulations suggest that *K^trans^* is considerably more predictive of both outcomes than the other three parameters.

**References**

Venkatasubramanian R, Henson MA, Forbes NS (2006) Incorporating energy metabolism into a growth model of multicellular tumor spheroids. *J Theor Biol* **242**(2)**:** 440-53

Venkatasubramanian R, Henson MA, Forbes NS (2008) Integrating cell-cycle progression, drug penetration and energy metabolism to identify improved cancer therapeutic strategies. *J Theor Biol* **253**(1)**:** 98-117

William D. McArdle FIK, Victor L. Katch (2006) Gas Excahnge and transport. In *Exercise physiology: energy, nutrition, and human performance*, 6, illustrated edn, p 1068. Lippincott Williams & Wilkins
